# 

**Published:** 1902-02

**Authors:** 


The Texas State Medical Association will hold its thirty-fourth annual meeting in Dallas, beginning May 6th, and continuing four days. Details of arrangements and all particulars will in due time be published in the Journal.

The American Congress of Tuberculosis.—The third annual session of this congress is announced to be held on the 14th, 15th and 16th of May, 1902, in the city of New York, in joint session with the Medico-Legal Society. There will be two sessions each day and no evening session, except on the 15th, when the banquet will be given. This will enable delegates from distant States and countries to enjoy the amusements and attractions of the city.

Arrangements will be made with railway companies for a reduced rate of fare, the details of which will be announced to the delegates.

There will be, aside from all papers of a miscellaneous nature, four symposiums, arranged each to occupy one session of the body, viz.:

1. Preventive Legislation, Embracing the Social, Municipal, and State Aspects of Tuberculosis.
2. Tuberculosis in its Pathological and Bacteriological Aspects.

3. The Medical and Surgical Aspects of Tuberculosis.
4. The Veterinary Aspects of Tuberculosis.

These will each be in charge of a committee who will arrange for the opening papers, and for those who participate. These committees will be arranged with great care and duly announced.

All medical bodies, and scientific or legal associations, or associations of the bar, are invited to send delegates to the congress, who will be given the rights of the floor and a vote at the session.

The enrollment is open to members of both professions in every State, county or province on the continents of America, in the western hemisphere, and in American waters, and papers are promised and will be solicited from all who are interested, in foreign countries.

For details and enrollment, address Clark Bell, President, 39 Broadway, New York City.

[Judge Bell, at first secretary and man of all work, was elected and installed as president of the congress at the January meeting. —Ed.]

